# Alpha-Lipoic Acid Modulates Melanoma Survival Networks via ER Stress Induction, Mitochondrial Apoptosis, and Kinase Pathway Suppression in B16F10 Cells

**DOI:** 10.3390/cimb48070690

**Published:** 2026-07-03

**Authors:** Ömer Kokaçya, Percin Pazarci, Halil Mahir Kaplan

**Affiliations:** 1 Department of Plastic, Reconstructive and Aesthetic Surgery, Faculty of Medicine, Cukurova University, 01330 Adana, Turkey; okokacya@cu.edu.tr; 2 Department of Medical Biology, Faculty of Medicine, Cukurova University, 01330 Adana, Turkey; percinpazarci@gmail.com; 3 Department of Pharmacology, Faculty of Medicine, Cukurova University, 01330 Adana, Turkey

**Keywords:** melanoma, alpha lipoic acid, apoptosis, cell survival, MAPK, ER stress

## Abstract

**Background/Objectives:** Malignant melanoma is characterized by constitutive PI3K/Akt/mTOR and MAPK activation, driving aggressive behavior and therapeutic resistance. Alpha-lipoic acid (αLA), a naturally occurring dithiol compound with an established clinical safety profile, has shown anticancer potential; however, its integrated molecular mechanisms in melanoma remain poorly defined. This study aimed to comprehensively evaluate the cytotoxic and mechanistic effects of αLA in B16F10 murine melanoma cells. **Methods:** Antiproliferative effects were assessed by MTT assay at four concentrations (250, 500, 750, 1000 µM) over 48 h. Protein levels of apoptotic markers (Bax, Bcl-2, Caspase-3, AIF), kinase signaling components (p-Akt, p-mTOR, p-ERK, p-JNK), ER stress markers (GRP78, GADD153/CHOP), and cell cycle regulator Wee1 were quantified by ELISA at a specifically selected sub-lethal concentration of 750 µM (inducing ~38% growth inhibition). **Results:** αLA dose-dependently inhibited B16F10 proliferation. At 750 µM, it triggered robust intrinsic apoptotic signaling, evidenced by a nearly 10-fold shift in the Bax/Bcl-2 ratio and greater than 9-fold Caspase-3 activation. Elevated AIF suggested profound mitochondrial stress and the potential priming of concurrent caspase-independent cell death mechanisms. αLA suppressed survival signaling by reducing p-Akt (44%), p-mTOR, p-ERK, and p-JNK. Treatment triggered lethal ER stress via GRP78 and GADD153/CHOP upregulation and upregulated Wee1, suggesting the induction of stress-responsive checkpoint signaling. The simultaneous CHOP upregulation and p-Akt suppression highlight a concurrent dysregulation of stress and survival pathways, suggesting a potential pro-apoptotic interplay. **Conclusions:** αLA exerts potent multi-target anticancer effects by inducing a broad spectrum of associated molecular changes, including the suppression of PI3K/Akt/mTOR and MAPK networks, induction of ER stress, engagement of cell cycle checkpoints, and activation of the mitochondrial Bax/Bcl-2/Caspase-3 axis. Importantly, these correlative findings do not establish proven pathway dependencies. Nevertheless, this concurrent dysregulation positions αLA as a potential disruptor of inter-pathway resilience underlying drug resistance.

## 1. Introduction

Malignant melanoma represents the most aggressive and lethal form of skin cancer, characterized by high metastatic potential and resistance to conventional chemotherapeutics [1]. Despite recent advances in targeted therapies and immunotherapies, the prognosis for advanced melanoma remains poor due to the complex network of survival signaling pathways that drive drug resistance [2]. In melanoma, oncogenic mutations—most notably in BRAF (predominantly the V600E substitution) and NRAS—lead to the constitutive and aberrant activation of the MAPK and PI3K/Akt/mTOR signaling cascades [3]. This persistent signaling environment not only drives uncontrolled cellular proliferation but also fosters a robust resistance to conventional therapeutic interventions [4]. Consequently, there is an urgent need to identify novel therapeutic agents that can effectively trigger cancer cell death by simultaneously targeting multiple molecular mechanisms, including apoptosis, cell cycle arrest, and stress response pathways. The B16F10 murine melanoma cell line was selected as the experimental model for this study due to its well-characterized high metastatic potential, constitutive activation of MAPK and PI3K/Akt/mTOR pathways, and its widespread use as a reliable and reproducible in vitro system for evaluating novel anticancer agents. Furthermore, the B16F10 model serves as a well-established preclinical bridge, as findings obtained in this system are readily translatable to syngeneic in vivo mouse models, facilitating future pharmacological validation.

Alpha-lipoic acid (αLA) is a naturally occurring dithiol compound widely known for its antioxidant properties. Notably, αLA is already approved for clinical use in the management of diabetic polyneuropathy and has demonstrated a favorable safety profile in human subjects, making it a particularly attractive candidate for oncological repurposing. Its ability to act as both a hydrophilic and lipophilic antioxidant, cross the blood–brain barrier, and regenerate endogenous antioxidants such as glutathione further supports its translational potential in cancer therapy [5,6]. Emerging evidence suggests that αLA can exert potent anti-cancer effects in various malignancies by inducing oxidative stress and modulating intracellular signaling [7,8]. While its cytotoxicity has been observed, the precise molecular machinery involving the interplay between ER stress, cell cycle regulation, and kinase signaling requires detailed elucidation in melanoma models. Although the general antiproliferative properties of αLA have been documented in various cancer models, its capacity to orchestrate a multi-pronged attack (specifically by integrating ER stress induction through GRP78/CHOP, cell cycle arrest via Wee1 modulation, and the simultaneous collapse of major kinase networks) remains significantly under-explored in melanoma. Our study addresses this gap by providing a comprehensive mechanistic framework for αLA’s action in the B16F10 model.

The Bcl-2 protein family serves as the primary regulator of programmed cell death, or apoptosis. Ultimately, the survival or demise of a cell hinges on the equilibrium between anti-apoptotic Bcl-2 and pro-apoptotic Bax. Perturbing this ratio causes permeabilization of the mitochondrial outer membrane, which sequentially triggers Caspase-3, the principal executioner enzyme [9]. Beyond this conventional caspase-mediated cascade, stress on the mitochondria can provoke the discharge of Apoptosis Inducing Factor (AIF). Once released, AIF moves into the nucleus, orchestrating DNA cleavage and cell death without relying on caspases [10]. Therefore, therapeutic interventions that concurrently engage both caspase-driven and independent mechanisms represent a powerful approach to eradicating malignant cells.

The survival and proliferation of melanoma cells are heavily reliant on the PI3K/Akt/mTOR and MAPK signaling pathways. Hyperactivation of Akt and its downstream effector mTOR promotes tumorigenesis and prevents apoptosis [11]. Similarly, the MAPK pathway, particularly through ERK phosphorylation, drives uncontrolled cell division, while JNK plays a dual role, often mediating stress-induced apoptotic signaling [12]. Therefore, the simultaneous inhibition of Akt, mTOR, and ERK, coupled with the modulation of JNK, represents a critical therapeutic objective. It is important to note that JNK signaling is context-dependent; while JNK activation is classically associated with stress-induced apoptosis, sustained or chronic JNK activity can paradoxically support tumor cell survival and contribute to chemoresistance [13]. Therefore, the net effect of JNK modulation must be interpreted within the broader signaling context of each specific cellular model and treatment condition.

Recent literature emphasizes the significance of Endoplasmic Reticulum (ER) stress in oncological treatments. As unfolded proteins build up, the Unfolded Protein Response (UPR) is triggered, evidenced by elevated levels of the GRP78 chaperone. Yet, when this stress becomes excessive or sustained, the UPR transitions from a protective mechanism to a lethal one by upregulating the pro-apoptotic transcription factor GADD153 (CHOP) [14]. Intimately connected to these stress mechanisms is cell cycle control. At the G2/M transition, Wee1 kinase acts as an essential checkpoint regulator. Altering Wee1 activity can either halt the cell cycle or induce mitotic catastrophe, thereby stopping the division of compromised cells [15]. Importantly, these stress-activated pathways do not operate in isolation. Accumulating evidence indicates that lethal ER stress can directly suppress the PI3K/Akt survival axis, partly through CHOP-mediated upregulation of PTEN, thereby establishing a pro-apoptotic feedback loop that reinforces the commitment to cell death [16]. This crosstalk between the ER stress response and kinase signaling networks represents a potentially critical node for therapeutic exploitation in treatment-resistant cancers such as melanoma.

In the present study, we aimed to investigate the multifaceted anticancer mechanisms of αLA in B16F10 melanoma cells. We comprehensively analyzed its effects on cytotoxicity and cell viability. Specifically, we examined the modulation of apoptotic markers (Bax, Bcl-2, Caspase-3, AIF), the regulation of key signaling pathways (p-Akt, p-mTOR, p-ERK, p-JNK), and the induction of ER stress and cell cycle arrest markers (GRP78, GADD153, Wee1). Our findings provide a holistic view of how αLA targets these interconnected pathways to suppress melanoma progression.

## 2. Materials and Methods

### 2.1. Cell Culture and Treatments

Murine B16F10 melanoma cells, obtained from the American Type Culture Collection (ATCC, Manassas, VA, USA), were cultured in Dulbecco’s Modified Eagle Medium (DMEM, Thermo Fischer, Waltham, MA, USA). This medium was enriched with 1% penicillin/streptomycin and 10% heat-inactivated fetal bovine serum (FBS). Incubation was carried out at 37 °C within a humidified 5% CO_2_ environment [17]. To formulate stock solutions, αLA was freshly dissolved in dimethyl sulfoxide (DMSO, Thermo Fischer, Waltham, MA, USA) immediately prior to each experiment to prevent time-dependent solvent-solute interactions. Careful regulation was maintained to keep the final solvent concentration strictly below 0.1% in all assays. For all experimental procedures, a vehicle control group comprising the culture medium and DMSO was evaluated in parallel.

### 2.2. Cell Viability Assay (MTT)

The 3-(4,5-dimethylthiazol-2-yl)-2,5-diphenyltetrazolium bromide (MTT, Thermo Fischer, Waltham, MA, USA) assay was utilized to assess the cytotoxicity of αLA against B16F10 cells. After seeding the cells in 96-well plates and allowing overnight attachment, the initial medium was substituted with fresh medium supplemented with various αLA concentrations (250, 500, 750, and 1000 µM). Following a 48 h exposure, the treatment medium was removed, and 5 mg/mL of MTT reagent was introduced into the wells. The plates were then kept in the dark for a 4 h incubation period. Dimethyl sulfoxide (DMSO) was used to dissolve the formed formazan crystals, and a microplate reader (Rayto, RT-2100C, Shenzhen, China) recorded the absorbance values at 570 nm. Cell viability was determined as a percentage compared to the untreated control [18]. Consequently, the viability data indicated that 750 µM was the ideal working concentration, which was subsequently chosen for all downstream ELISA evaluations.

### 2.3. Preparation of Cell Lysates and Protein Quantification

After treating the B16F10 cells with the predetermined 750 µM dose of αLA, they were collected for protein expression evaluation. To eliminate any remaining culture medium, the cells underwent two wash cycles with ice-cold phosphate-buffered saline (PBS). Lysis was then carried out using a radioimmunoprecipitation assay (RIPA) buffer, which was enriched with a cocktail of protease and phosphatase inhibitors. Complete cellular disruption was achieved through ultrasonication on ice. Following this, the homogenized samples were subjected to centrifugation at 15,000 rpm and 4 °C for 20 min to separate the debris. The resulting supernatants were harvested, and total protein yields were quantified using the Bradford assay protocol [17].

### 2.4. Assessment of Signaling Pathways via ELISA

The levels of specific proteins were quantified using ELISA kits (Sun-Red Bio Inc., Shanghai, China), strictly adhering to the manufacturer’s protocols. To provide a comprehensive understanding of the molecular mechanisms underlying the effects of αLA, the analysis focused on three key aspects. First, to assess the balance between cell death and survival, the expression levels of Bax, Bcl-2, Caspase-3 and AIF were measured. Concurrently, the impact on major intracellular signaling routes involved in proliferation was evaluated by quantifying p-Akt, p-mTOR, p-ERK, and p-JNK levels. Finally, to investigate the mechanisms of cellular stress and cycle arrest, the expression levels of GRP78, GADD153 and Wee1 were determined.

Because the absolute quantification units (e.g., pg/mL, ng/mL, U/mL) are defined by the specific manufacturer standards for each target protein kit, direct stoichiometric comparisons of absolute concentrations between different proteins cannot be made. Therefore, the data were analyzed and interpreted based on the relative expression changes (fold-change or percentage differences) between the untreated control and αLA-treated states for each individual marker.

### 2.5. Statistical Analysis

Quantitative results are presented as the mean ± standard error of the mean (SEM), reflecting data gathered from six independent biological replicates (*n* = 6, utilizing distinct cell passages). Because the study evaluated a targeted set of pre-defined molecular markers based on a priori hypotheses, and comparisons were strictly limited to two distinct groups (untreated control versus αLA-treated), an unpaired Student’s *t*-test was deemed the appropriate statistical method to evaluate differences. GraphPad Prism software (version 9.0) was utilized for both statistical evaluation and graphical representation. To rigorously control for potential false-positive findings within this targeted panel, statistical significance was stringently defined a priori as a *p*-value below 0.01 (*p* < 0.01). All the experimental data used in statistical analysis is given as Supplementary Materials (Tables S1–S10).

## 3. Results

### 3.1. Cytotoxic Effects of αLA on B16F10 Melanoma Cells

**Figure 1:**
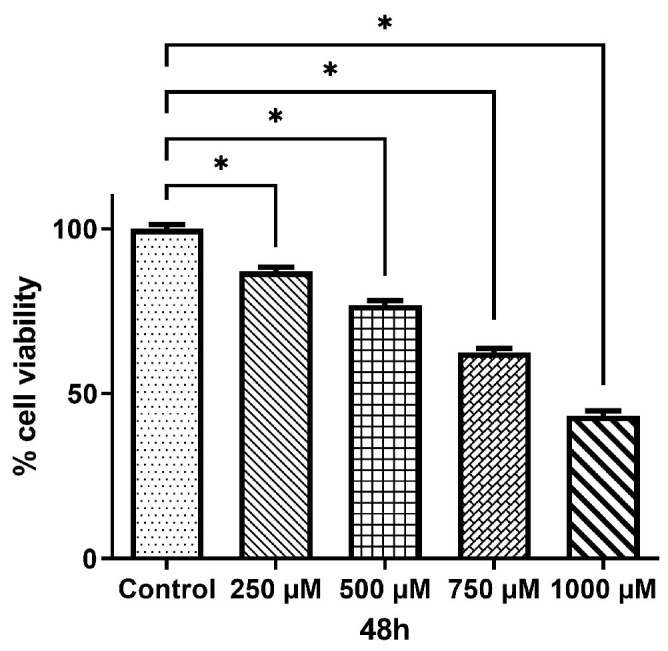
Evaluation of αLA-induced cytotoxicity in B16F10 melanoma cells. The MTT assay was utilized to assess cell viability. Results are expressed as the mean ± SEM of six independent biological replicates (*n* = 6) (*: *p* < 0.01).

The effect of αLA on the proliferation of B16F10 cells was investigated using the MTT assay. The cells were treated with various concentrations of αLA (250, 500, 750, and 1000 µM) for 48 h. As shown in Figure 1, αLA treatment caused a significant and dose-dependent reduction in cell viability compared to the control group (*p* < 0.01 for all concentrations). While the control group exhibited 100% viability, exposure to 250, 500, 750, and 1000 µM αLA decreased the viability to 86.91% ± 1.403, 76.86% ± 1.279, 62.40% ± 1.388, and 43.23% ± 1.558, respectively. Formal nonlinear regression analysis of this data estimated the true IC50 value at approximately 923 µM. Based on these findings, rather than utilizing the formal IC50, the sub-lethal 750 µM concentration—which specifically inhibited cell growth by approximately 38%—was deliberately selected as the optimal dose. This sub-IC50 concentration robustly triggers stress and apoptotic cascades without the confounding interference of severe, nonspecific secondary necrosis, making it ideal for further precise mechanistic studies.

### 3.2. αLA Activates Apoptotic Signaling via Modulation of Bax, Bcl-2 and Caspase-3

**Figure 2:**
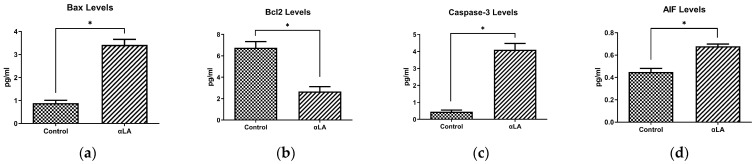
Effect of αLA treatment on apoptosis-related markers. (**a**) Bax. (**b**) Bcl-2. (**c**) Cleaved Caspase-3. (**d**) AIF. Results are expressed as the mean ± SEM of six independent biological replicates (*n* = 6) (*: *p* < 0.01).

To investigate if apoptosis mediated the growth-suppressive impact of αLA, we quantified vital apoptotic indicators via ELISA in B16F10 cells exposed to 750 µM αLA. Our findings demonstrated profound shifts in Bcl-2 family protein concentrations. Specifically, the αLA-exposed cells exhibited a marked, approximately 3.8-fold elevation in pro-apoptotic Bax levels (3.417 ± 0.241 pg/mL) relative to the untreated controls (0.888 ± 0.122 pg/mL; *p* < 0.01) (Figure 2a). In contrast, αLA application notably suppressed the anti-apoptotic Bcl-2 protein, decreasing its concentration from 6.767 ± 0.567 pg/mL in controls to 2.667 ± 0.460 pg/mL in the treated group/cells (*p* < 0.01) (Figure 2b). This pro-apoptotic disruption of the Bax/Bcl-2 ratio was corroborated by Caspase-3 assessments; the concentration of cleaved Caspase-3 surged dramatically by over 9-fold following αLA treatment (4.100 ± 0.372 pg/mL) versus the control (0.452 ± 0.095 pg/mL; *p* < 0.01) (Figure 2c), signaling active apoptosis. Furthermore, AIF concentrations were significantly upregulated in the treated cells (0.68 ± 0.02 pg/mL) compared to controls (0.45 ± 0.03 pg/mL), pointing toward mitochondrial stress or caspase-independent apoptotic mechanisms (Figure 2d).

### 3.3. αLA Downregulates p-Akt, p-mTOR, p-ERK, and p-JNK Levels

**Figure 3:**
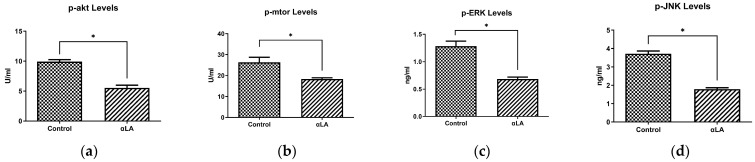
Inhibition of PI3K/Akt/mTOR and MAPK signaling pathways by αLA. (**a**) p-Akt. (**b**) p-mTOR. (**c**) p-ERK. (**d**) p-JNK. Results are expressed as the mean ± SEM of six independent biological replicates (*n* = 6) (*: *p* < 0.01).

To investigate the molecular mechanisms underlying the apoptotic and cytotoxic effects of αLA, the phosphorylation status of key survival- and proliferation-associated proteins was analyzed using ELISA. The results demonstrated that αLA treatment significantly suppressed the PI3K/Akt/mTOR signaling axis. The levels of p-Akt were significantly reduced by approximately 44% in the αLA-treated group (5.533 ± 0.444 U/mL) compared to the control group (9.933 ± 0.328 U/mL; *p* < 0.01) (Figure 3a). Furthermore, a significant downregulation of p-mTOR was observed in the αLA group (18.33 ± 0.615 U/mL) compared to the control (26.33 ± 2.431 U/mL; *p* < 0.01) (Figure 3b). In addition to the Akt pathway, the MAPK signaling pathway components were also evaluated. p-ERK levels were found to be significantly lower in αLA-treated cells (0.683 ± 0.036 ng/mL) compared to the control cells (1.283 ± 0.091 ng/mL; *p* < 0.01) (Figure 3c). Similarly, p-JNK levels exhibited a highly significant 52% decrease following αLA treatment (1.793 ± 0.075 ng/mL) compared to the control (3.717 ± 0.147 ng/mL; *p* < 0.01) (Figure 3d). These results collectively suggest that αLA exerts its anticancer effects by simultaneously targeting the Akt/mTOR and MAPK signaling pathways.

### 3.4. αLA Upregulates GRP78, GADD153 and Wee1 Levels

To gain further insights into the stress response pathways triggered by αLA, the expression levels of GRP78, GADD153 (ER stress markers) and Wee1 (cell cycle regulator) were evaluated. The results indicated that αLA treatment induced a significant stress response in B16F10 cells, as detailed in Table 1.

The levels of the ER stress sensor GRP78 were significantly upregulated in the αLA-treated group (0.98 ± 0.06 pg/mL) compared to the control group (0.52 ± 0.05 pg/mL). Similarly, the expression of GADD153 (CHOP), a key mediator of ER stress-induced apoptosis, was markedly increased by 2.5-fold, rising from 0.35 ± 0.03 pg/mL in the control group to 0.88 ± 0.05 pg/mL following αLA treatment. Furthermore, the analysis showed a significant elevation in Wee1 levels, a critical regulator of the G2/M cell cycle checkpoint, in the treated group (0.58 ± 0.03 pg/mL) compared to the control (0.30 ± 0.02 pg/mL) (Table 1).

## 4. Discussion

Malignant melanoma remains one of the most aggressive and treatment-resistant forms of skin cancer, necessitating the development of novel therapeutic agents that can simultaneously target multiple oncogenic pathways [2]. In the present study, we investigated the anticancer potential of αLA on the B16F10 melanoma cell line. Our findings provide compelling evidence that αLA exerts a potent cytotoxic effect by triggering apoptosis, inducing ER stress, arresting the cell cycle, and suppressing key survival signaling pathways, specifically PI3K/Akt/mTOR and MAPK.

Preliminary evaluation of cell survival via the MTT method demonstrated that αLA suppresses B16F10 cell growth in a dose-dependent fashion. This cytotoxic behavior is consistent with prior literature documenting the anti-proliferative actions of αLA across multiple malignancies, such as colon, lung, and breast cancers [8,19,20]. Nevertheless, elucidating the specific cell death mechanism is essential for evaluating therapeutic potential. Our ELISA evaluations established that this cytotoxicity was mediated by apoptosis, the most favorable cell death route in oncological treatments. The notable suppression of anti-apoptotic Bcl-2, alongside the concurrent elevation of pro-apoptotic Bax, signifies the triggering of the intrinsic mitochondrial pathway. Quantitatively, the Bax/Bcl-2 ratio shifted from approximately 0.13 in control cells to 1.28 following αLA treatment (nearly 10-fold increase) a magnitude that strongly favors mitochondrial outer membrane permeabilization and apoptosome formation. This dramatic shift in the apoptotic rheostat is particularly noteworthy given that the selected 750 µM concentration, which corresponds to the approximate IC50 at 48 h, achieved this profound pro-apoptotic reprogramming without inducing complete necrotic cytotoxicity. While the 750 µM concentration may appear elevated compared to conventional synthetic chemotherapeutics, it is highly consistent with the functional in vitro ranges reported for natural metabolic modulators like αLA [21]. Although αLA is a physiologically tolerated molecule with an established clinical safety profile in humans, the presumed resilience of normal cells to this relatively high in vitro concentration remains to be experimentally confirmed in our single murine cell line model. In contrast, melanoma cells, which operate under heightened basal metabolic and endoplasmic reticulum stress, are specifically vulnerable to this targeted disruption [22]. This critical modification in the Bax/Bcl-2 balance promotes the discharge of apoptogenic mediators, which subsequently activate the executioner enzyme, Caspase-3 [9,23]. The prominent surge in cleaved Caspase-3 concentrations observed in our research indicates the robust activation of apoptotic executioner signaling in αLA-exposed B16F10 cells. Additionally, the elevated total AIF expressions imply that αLA induces severe mitochondrial breakdown [10,24]. While this suggests the potential priming of caspase-independent apoptotic pathways, the lack of functional nuclear translocation data means this specific execution axis remains to be definitively confirmed. These apoptotic findings are consistent with, yet extend beyond, previous reports in other malignancies. A previous study demonstrated that αLA induces apoptosis in colon cancer cells through mitochondrial respiration-coupled oxidative stress, while another study reported synergistic pro-apoptotic effects of αLA combined with radiotherapy in breast cancer via HMGB1-mediated pathways [8,19]. In contrast to these single-mechanism observations, our data in B16F10 cells reveal a convergent activation of both caspase-dependent (Caspase-3) and caspase-independent (AIF) apoptotic arms, suggesting that αLA orchestrates a broader and potentially more robust apoptotic program in melanoma than previously described in other tumor types.

To elucidate the upstream mechanisms governing these apoptotic events, we analyzed the PI3K/Akt/mTOR signaling axis, which is frequently hyperactivated in melanoma and associated with drug resistance and survival [11,25,26]. αLA treatment resulted in a marked suppression of p-Akt and its downstream effector p-mTOR. The inhibition of this pathway is crucial, as Akt promotes cell survival by inhibiting pro-apoptotic proteins (such as Bax) and activating anti-apoptotic ones [27,28]. The observed reductions in Akt/mTOR phosphorylation suggest that αLA may compromise the survival signals that protect melanoma cells, reflecting associated molecular changes rather than the definitive deactivation of these entire networks.

Concurrently, we observed a significant downregulation in the MAPK pathway components, specifically p-ERK and p-JNK. The ERK pathway is a central driver of cell proliferation and differentiation, and its inhibition is a primary target in melanoma therapy [29,30]. The concurrent reduction in p-ERK levels by αLA further corroborates its antiproliferative activity. Interestingly, while JNK activation is often associated with stress-induced apoptosis, it can also support cell survival and tumorigenesis depending on the context [31,32]. The interpretation of p-JNK reduction warrants careful consideration, as JNK signaling is inherently context-dependent. In many tumor models, acute JNK activation mediates stress-induced apoptosis; however, chronic or sustained JNK activity has been increasingly recognized as a driver of tumor cell survival, resistance to chemotherapy, and pro-tumorigenic inflammation [13]. In the B16F10 model, where constitutive MAPK activation underpins survival signaling, the observed reduction in p-JNK by αLA may reflect the dismantling of a pro-survival, rather than pro-apoptotic, arm of JNK activity [3]. This interpretation is supported by the concurrent robust activation of the apoptotic machinery (elevated Bax, Caspase-3, AIF), which indicates that apoptotic commitment proceeded through mitochondrial and ER stress pathways independently of JNK activity. Nevertheless, we acknowledge that the precise temporal dynamics of JNK modulation (whether an early transient activation preceded the sustained suppression observed at 48 h) could not be resolved within the single time-point design of the present study, and this remains a question for future investigation.

A novel aspect of our study involves the role of ER stress and cell cycle regulation. We found that αLA treatment significantly upregulated GRP78 and GADD153 (CHOP). GRP78 is a chaperone that acts as a sensor for ER stress; its elevation indicates the accumulation of unfolded proteins [33]. Prolonged or severe ER stress leads to the induction of GADD153, a transcription factor that links ER stress to apoptosis by downregulating Bcl-2 and upregulating Bax [34]. Our data suggest that αLA induces lethal ER stress (Unfolded Protein Response), contributing to the apoptotic outcome. However, we explicitly caution that the isolated elevation of GRP78 and CHOP does not constitute comprehensive evidence for the global activation of the major Unfolded Protein Response (UPR) branches (such as PERK, IRE1α, and ATF6), as these specific upstream pathways were not directly examined. A particularly noteworthy finding in our study is the potential cross-talk between the induced ER stress and the suppression of survival signaling. Elevated levels of GADD153 (CHOP) have been shown to negatively regulate the PI3K/Akt pathway, effectively creating a pro-apoptotic feedback loop that ensures the commitment to cell death [35]. The simultaneous upregulation of CHOP and the dramatic decline in p-Akt levels in αLA-treated B16F10 cells suggest that αLA concurrently modulates these major pathways. Drawing upon existing literature, we hypothesize that this simultaneous modulation might involve the ER-mitochondria-signaling junction [36]. Mechanistically, it is plausible that this interplay is mediated through CHOP-driven transcriptional upregulation of PTEN, the primary negative regulator of the PI3K/Akt axis: αLA-induced ER stress activates CHOP, which suppresses Akt survival signaling, which in turn lowers the apoptotic threshold and sensitizes cells to the Bax/Caspase-3 cascade [16]. While our data demonstrate the co-occurrence of these events, further genetic validation is required to definitively establish a self-reinforcing pro-apoptotic circuit in this specific model. This integrated model positions αLA not merely as an inhibitor of individual pathways, but as a disruptor of the inter-pathway communication network that melanoma cells rely upon for therapeutic resistance. Moreover, the significant increase in Wee1, a tyrosine kinase that regulates the G2/M transition, indicates that αLA treatment engages this critical cell cycle checkpoint. As astutely noted in the recent literature, elevated Wee1 initially serves as a compensatory, protective response, preventing entry into mitosis to allow time for cellular repair. However, we strictly acknowledge that Wee1 expression alone provides limited biological insight into actual cell cycle dynamics. Without accompanying flow cytometric cell-cycle analysis, the definitive functional distribution of the cells cannot be determined. In the specific context of our findings—where survival signals (Akt/mTOR) are profoundly suppressed and lethal ER stress (CHOP) is simultaneously activated—this protective checkpoint is likely overwhelmed. Consequently, the inability to resolve the catastrophic stress ultimately drives the B16F10 cells toward the observed Caspase-3-mediated apoptosis [19,37].

While our results provide a robust mechanistic framework for the anticancer activity of αLA, several limitations inherent to the study design should be acknowledged. First, this study was conducted using an in vitro B16F10 murine melanoma model, which inherently fails to encapsulate the complex tumor microenvironment. More critically, the 750 µM concentration utilized for mechanistic evaluations, while optimal for overcoming the rapid compound oxidation and the artificially high basal proliferation rates of 2D cultures, exceeds standard achievable human plasma concentrations. This discrepancy highlights a significant gap in systemic pharmacokinetics, such as the metabolic half-life, tissue accumulation, and bioavailability of αLA in vivo. Therefore, direct translational interpretability is limited, and future in vivo models are strictly required to determine clinically plausible, dose-equivalent efficacies. Second, all protein quantifications were performed at a single time point (48 h) and exclusively at a single targeted concentration (750 µM). While this specific dose was empirically selected to capture active molecular signaling prior to the onset of overwhelming non-specific cytotoxicity, the lack of a multi-dose molecular assessment precludes our ability to determine whether the observed signaling changes scale proportionally with decreased viability or represent a discrete biological threshold effect. Consequently, precluding the characterization of temporal signaling dynamics (particularly the early versus late phases of JNK modulation and ER stress activation) and dose-dependent network responses limits a complete mechanistic understanding. Crucially, while the robust upregulation of the master sensor GRP78 and the pro-apoptotic executioner CHOP confirms the engagement of lethal ER stress, the specific upstream branches of the Unfolded Protein Response (UPR)—namely the PERK–eIF2α–ATF4 axis, IRE1α activation and XBP1 splicing, and ATF6 signaling—were not individually characterized. Future temporal and comprehensive dose–response pathway analyses are required to fully elucidate the complete mechanistic dynamics. Third, protein expression was assessed exclusively via ELISA. While samples were strictly normalized for total protein concentration using the Bradford assay prior to analysis, the lack of phosphorylated-to-total protein ratios (e.g., p-Akt/total Akt) and independent corroboration via orthogonal methods such as Western blotting or immunofluorescence represents a notable limitation. Although the simultaneous robust upregulation of apoptotic and stress markers (Bax, Caspase-3, CHOP) alongside the downregulation of kinases strongly suggests targeted pathway suppression rather than non-specific protein degradation, future studies must validate these specific kinase activation states using total protein ratios. Additionally, corroborating mRNA expression data through qRT-PCR would further strengthen the mechanistic conclusions drawn from these findings. Fourth, the lack of orthogonal cytometric and imaging analyses—such as Annexin V/PI flow cytometry to precisely quantify apoptotic populations, and DNA content staining combined with the assessment of canonical regulators (e.g., cyclin B1, CDK1 phosphorylation status) to definitively confirm cell cycle distribution—limits our ability to definitively map these specific cellular fates. While the elevation of total AIF indicates robust mitochondrial stress, the lack of subcellular fractionation or immunofluorescence assays to confirm its functional nuclear translocation prevents definitive conclusions regarding the actual execution of caspase-independent apoptosis. Future studies must incorporate these comprehensive methodologies to validate the proposed checkpoint engagements. Fifth, a significant limitation of the present study is the absence of parallel cytotoxicity assessments on non-cancerous control cell lines, such as normal melanocytes or dermal fibroblasts. Consequently, an exact in vitro selectivity index for αLA in this specific experimental setup could not be mathematically established. The established clinical safety profile of αLA cannot substitute for direct experimental assessment of differential cytotoxicity in this model. Future preclinical investigations must include healthy cell models to rigorously quantify the therapeutic window of αLA before advancing to extensive in vivo testing. Sixth, while we observed the simultaneous upregulation of stress markers (e.g., CHOP, Caspase-3) and the suppression of survival kinases (e.g., Akt, ERK), all mechanistic relationships were inferred from static endpoint measurements. The lack of direct functional validation—such as gain- or loss-of-function genetic perturbations (e.g., CHOP or PTEN knockdown), target rescue approaches (e.g., Akt activation), or the use of specific pharmacological inhibitors (e.g., pan-caspase inhibitors)—means that definitive causal relationships and absolute pathway dependencies cannot be confirmed. The proposed network interplays remain correlative hypotheses that necessitate rigorous functional validation in future studies. Seventh, the present study did not include a standard chemotherapeutic reference agent (such as dacarbazine or temozolomide) as a positive control. Consequently, the relative potency and magnitude of αLA’s effects compared to established first-line melanoma treatments could not be directly contextualized. Since αLA effectively targets the survival networks (such as PI3K/Akt/mTOR and MAPK) that often mediate chemoresistance, future investigations should not only benchmark αLA against standard chemotherapeutics but also explore their combined application to evaluate potential synergistic or chemo-sensitizing effects. Eighth, while DMSO concentration was carefully controlled below 0.1%, the potential interaction between αLA’s reactive dithiol group and DMSO under cell culture conditions, though unlikely at this concentration, represents a minor confounding variable that warrants acknowledgment. Finally, the exclusive use of a murine cell line limits the direct extrapolation of these findings to human melanoma biology. Consequently, we explicitly acknowledge that our current findings serve as a fundamental mechanistic proof-of-concept, and their direct translational relevance remains substantially restricted. Validation in human-derived melanoma cell lines harboring clinically relevant BRAF V600E mutations, such as A375 or SK-MEL-28, alongside in vivo xenograft models, represents an essential and logical next step toward translating these findings into a clinical context.

## 5. Conclusions

The present study demonstrates that alpha-lipoic acid (αLA) exerts potent and multifaceted anticancer effects against B16F10 murine melanoma cells through the simultaneous engagement of several interconnected oncogenic pathways. At the cellular level, αLA induced a significant dose-dependent reduction in cell viability, with a nearly 10-fold shift in the Bax/Bcl-2 ratio and robust activation of Caspase-3, confirming the activation of intrinsic mitochondrial apoptotic signaling cascades. The concurrent elevation of total AIF levels highlights severe mitochondrial stress and suggests the potential parallel involvement of caspase-independent cell death mechanisms, collectively underscoring the breadth of the apoptotic program triggered by αLA.

At the signaling level, αLA comprehensively suppressed the PI3K/Akt/mTOR and MAPK axes—two survival networks that are constitutively activated in melanoma and intimately linked to therapeutic resistance. The simultaneous downregulation of p-Akt, p-mTOR, p-ERK, and p-JNK suggests that αLA concurrently modulates broader kinase networks associated with melanoma cell survival. Furthermore, the co-occurrence of CHOP-mediated ER stress and Akt suppression highlights a potential interplay between stress induction and survival inhibition. We explicitly note that our static endpoint data identify associated molecular changes rather than proven causal mechanisms. Because no functional perturbation experiments (such as genetic knockdown or specific pharmacological inhibition) were conducted, definitive pathway dependencies cannot be established from this study.

Taken together, these correlative findings establish a mechanistic proof-of-concept that αLA can simultaneously modulate multiple survival and stress pathways in a murine melanoma model at a high, sub-lethal in vitro concentration (750 µM). However, given the lack of differential cytotoxicity data, human cell line validation, and the uncertainty regarding the clinical achievability of the utilized concentration, the direct translational relevance of these findings is currently restricted. αLA warrants rigorous future investigation in human-derived melanoma cell lines and in vivo models to establish its true translational potential and explore its hypothetical utility as an adjuvant agent.

## Figures and Tables

**Table 1:** Effect of αLA treatment on the levels of ER stress and cell cycle regulatory proteins in B16F10 cells.

	Control	αLA
Wee1	0.30 ± 0.02 pg/mL	0.58 ± 0.03 pg/mL *
GADD153	0.35 ± 0.03 pg/mL	0.88 ± 0.05 pg/mL *
GRP78	0.52 ± 0.05 pg/mL	0.98 ± 0.06 pg/mL *

Results are expressed as the mean ± SEM of six independent biological replicates (*n* = 6). *: *p* < 0.01 indicates a significant difference compared to the Control group.

## Data Availability

The original contributions presented in this study are included in the article/Supplementary Materials. Further inquiries can be directed to the corresponding author.

## Supplementary Materials

The following supporting information can be downloaded at: https://www.mdpi.com/article/10.3390/cimb48070690/s1.
