# Supplementary material for: Alpha-Lipoic Acid Modulates Melanoma Survival Networks via ER Stress Induction, Mitochondrial Apoptosis, and Kinase Pathway Suppression in B16F10 Cells
Source: Curr Issues Mol Biol. 2026 Jul 3;48(7):690. doi: 10.3390/cimb48070690 (PMC13407186; doi:10.3390/cimb48070690)
Supplement: Supplementary file 1 [file cimb-48-00690-s001.zip › cimb-4413325-supplementary.pdf]

Table S1. Data corresponding to Figure 1.

MTT results				
**Control**	**250 μM**	**500 μM**	**750 μM**	**1000 μM**
110	90	88	72	50
108	95	85	65	48
111	99	88	72	45
108	100	80	70	40
105	93	81	68	51
115	94	83	63	50

Table S2. Data corresponding to Figure 1 (normalized data).

MTT results normalized to 100%				
**Control**	**250 μM**	**500 μM**	**750 μM**	**1000 μM**
100.45705	82.191781	80.365297	65.753425	45.6621
98.630137	86.757991	77.625571	59.360731	43.835616
101.36986	90.410959	80.365297	65.753425	41.09589
98.630137	91.324201	73.059361	63.926941	36.52968
95.890411	84.931507	73.972603	62.100457	46.575342
105.02283	85.844749	75.799087	57.534247	45.6621

Table S3. Data corresponding to Figure 2 (Bax).

Bax	
**Control**	**αLA**
0.6	2.5
1.2	3.2
0.55	4.1
0.75	4
1	3.2
1.23	3.5

Table S4. Data corresponding to Figure 2 (Bcl2).

Bcl2	
**Control**	**αLA**
7.5	3.8
8.5	3.5
7.8	3.5
4.8	2.2
6	2.1
6	0.9

Table S5. Data corresponding to Figure 2 (Caspase-3).

Caspase-3	
Control	αLA
0.11	4.2
0.5	4.5
0.5	5.1
0.8	4.8
0.5	3.2
0.3	2.8

Table S6. Data corresponding to Figure 2 (AIF).

AIF	
Control	αLA
0.35	0.61
0.39	0.64
0.45	0.67
0.45	0.69
0.51	0.71
0.55	0.75

Table S7. Data corresponding to Figure 3 (p-akt).

p-akt	
Control	αLA
10	5.1
11	5
9	5.6
9	4
10.1	7
10.5	6.5

Table S8. Data corresponding to Figure 3 (p-mtor).

p-mtor	
Control	αLA
25	18
22	20
38	16
26	20
22	18
25	18

Table S9. Data corresponding to Figure 3 (p-ERK).

p-ERK	
**Control**	**αLA**
1.1	0.7
1.1	0.65
1.5	0.55
1.3	0.65
1.6	0.75
1.1	0.8

Table S10. Data corresponding to Figure 3 (p-JNK).

p-JNK	
**Control**	**αLA**
4	2
4.2	1.8
3.8	1.9
3.5	1.5
3.6	1.66
3.2	1.9
